# Influence of the heteroatom on the optoelectronic properties and transistor performance of soluble thiophene-, selenophene- and tellurophene–vinylene copolymers†
†Electronic supplementary information (ESI) available: Transistor transfer and output plots and PESA plots. See DOI: 10.1039/c5sc03501e


**DOI:** 10.1039/c5sc03501e

**Published:** 2015-11-02

**Authors:** Mohammed Al-Hashimi, Yang Han, Jeremy Smith, Hassan S. Bazzi, Siham Yousuf A. Alqaradawi, Scott E. Watkins, Thomas D. Anthopoulos, Martin Heeney

**Affiliations:** a Dept. Chemistry and Centre for Plastic Electronics , Imperial College London , Exhibition Rd , London , SW7 2AZ , UK . Email: m.heeney@imperial.ac.uk; b Dept. Chemistry , Texas A&M University at Qatar , P.O. Box 23874 , Doha , Qatar . Email: mohammed.al-hashimi@qatar.tamu.edu; c Dept. Physics and Centre for Plastic Electronics , Imperial College London , Exhibition Rd , London , SW7 2AZ , UK; d Dept. of Chemistry & Earth Sciences , Qatar University , P.O. Box 110003 , Doha , Qatar; e CSIRO , Molecular and Health Technologies , VIC 3169 , Australia

## Abstract

We report the first soluble poly(3-dodecyl tellurophenylene-vinylene) polymer (**P3TeV**) by Stille copolymerization and compare its properties to the analogous thiophene and selenophene containing polymers. The optical band gap of the polymers is shown to systematically decrease as the size of the heteroatom is increased, mainly as a result of a stabilization of the LUMO energy, resulting in a small band gap of 1.4 eV for **P3TeV**. Field effect transistors measurements in variety of architectures demonstrate that the selenophene polymer exhibits the highest mobility, highlighting that increasing the size of the heteroatom is not always beneficial for charge transport.

## Abstract 2

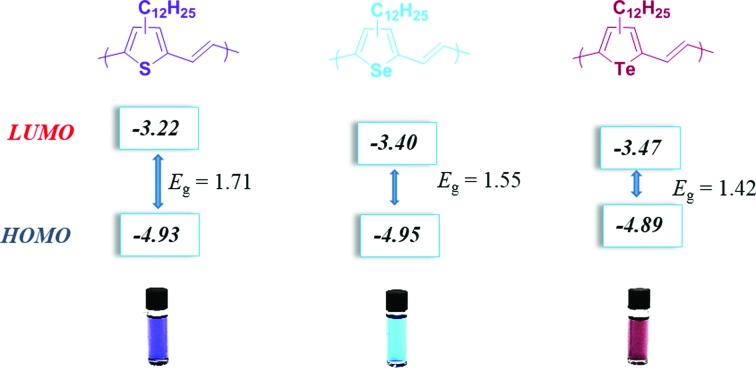
We report the first soluble poly(3-dodecyl tellurophenylenevinylene) and compare its properties to the analogous thiophene and selenophene containing polymers.

## Introduction

Conjugated organic polymers continue to attract widespread interest for a wide range of applications due to their potential for large area, low-cost fabrication.^1^,^2^ The ability of synthetic chemistry to manipulate the structure and properties of the conjugated aromatic monomers, as well as the almost limitless possibilities to co-polymerize such building blocks leads to a huge scope to tune the polymer performance for each application.^3^ Despite the large tool-box of organic chemistry, many of the polymers reported to date are based upon thiophene, or its fused ring analogues.^4^,^5^ One fascinating opportunity to manipulate the polymer properties is by changing the heteroatom in the five membered heterocycle from sulfur to other elements within group 16.^6^,^7^ Switching to heavier elements with larger polarizability such as selenium or tellurium is of potential interest since the larger atomic size may facilitate solid state intermolecular interactions, as well as allowing a route to control the HOMO and LUMO levels and optical band gap.^8^,^9^

Reports of tellurophene containing polymers are considerably less numerous than selenophene containing materials, probably in part due to the synthetic challenges of tellurophene chemistry.^10^,^11^ In addition many of the examples reported to date are co-polymers, with relatively low loadings of tellurophene in which there is a potential that the intrinsic influence of the heteroatom is masked.^12^–^20^ Homo-polymers of tellurophene are rare, with the first substituted tellurophene polymer, poly(3,4-dimethoxytellurophene),^21^ prepared as an insoluble film by electrochemical polymerization. A significant step was the recent report of the first soluble tellurophene polymer, poly(3-alkyltellurophene), P3ATe which was prepared by both electrochemical and Kumada catalyst transfer polymerization methods.^22^ Regioregular P3ATe exhibited a red shifted absorption compared to the thiophene or selenophene analogues, although the preliminary performance reported in solar cell was modest (PCE 1.1%).^23^

In this paper we report the synthesis and characterization of an analogous series of vinylene copolymers containing 3-dodecylthiophene, selenophene or tellurophene to allow the systematic investigation of the role of the heteroatom. 3-Alkylthiophene and 3-alkylselenophene–vinylene co-polymers have previously shown promise as the active semiconducting component in both FETs and OPVs,^24^,^25^ as well as in singlet fission applications.^26^ The tellurophene analogues have never been reported to the best of our knowledge. Promisingly the regioregularity of the alkyl chains has less influence on crystallinity in these systems than the non-vinylene co-polymers, due to the reduction of torsional disorder with the vinylene spacer.^27^ Herein, we report the synthesis of a novel 2,5-dibrominated 3-dodecyltellurophene monomer and its copolymerization with (*E*)1,2-bis(tributylstannyl)ethylene to afford poly(3-dodecyl-2,5-tellurophenylenevinylene) (**P3TeV**). We compare the optoelectronic properties to the analogous thiophene (**P3TV**) and selenophene (**P3SV**) containing polymers, as well as report the properties of all three polymer organic field-effect transistors (OFETs).

## Experimental

### General procedures

All chemicals were purchased from Aldrich or Fisher. All reactions were carried out under argon using solvents and reagents as commercially supplied, unless otherwise stated. ^1^H and ^13^C NMR spectra were recorded on a Bruker AV-400 (400 MHz), using the residual solvent resonance of CDCl_3_ or TMS as an internal reference and are given in ppm. Microwave reactions were run in a Biotage Initiator at constant temperature. Number-average (*M*_n_) and weight-average (*M*_w_) were determined by Agilent Technologies 1200 series GPC running in chlorobenzene at 80 °C. The system used two PL mixed B columns in series, and were calibrated against narrow polydispersity polystyrene standards. Electrospray mass spectrometry was performed with a Thermo Electron Corporation DSQII mass spectrometer. UV-Vis spectra were recorded on a Perkin Elmer Lambda 900 UV-Vis spectrometer. Flash chromatography (FC) was performed on silica gel (Merck Kieselgel 60 F254 230–400 mesh) unless otherwise indicated. Thin Layer Chromatography (TLC) was performed on Merck aluminium-backed plates pre-coated with silica (0.2 mm, 60 F254). Photo Electron Spectroscopy in Air (PESA) measurements were recorded with a Riken Keiki AC-2 PESA spectrometer with a power setting of 5 nW and a power number of 0.5. Samples for PESA were prepared on glass substrates as drop cast films. 3-Dodecylselenophene was prepared according to the published procedure.^28^

#### 3-Dodecyltellurophene

[**Caution**: some tellurium containing compounds are highly toxic and care should be taken when handling them]. To a 3 necked flask, fitted with a reflux condenser under argon was added tellurium powder (5.8 g, 45.45 mmol) and sodium borohydride granules (2.04 g, 53.97 mmol) and the mixture was stirred. The flask was cooled to 0 °C and ethanol (60 mL) was added dropwise over 1 h, maintaining the temperature below 20 °C. After complete addition the mixture was stirred at 0 °C for 20 min. To this was added a solution of 3-chloromethyl-1-pentadecyn-3-ol (7 g, 25.66 mmol) in ethanol (20 mL) dropwise over 20 min. After complete addition the mixture was stirred at 0 °C for 40 min and a solution of potassium hydroxide (2.55 g, 45.5 mmol) in ethanol (70 mL) was added over 10 min. The reaction mixture was refluxed for 2 h to give a brown solution. The mixture was allowed to cool to room temperature, and poured into ice/water (100 mL), the mixture was extracted with DCM (3 × 100 mL). The combined organic extracts were washed with brine (100 mL), dried over MgSO_4_, filtered and the solvent removed under reduced pressure to afford a yellow oil. To this hexane (100 mL) and *p*-toluene sulfonic acid (24 mg) were added. The mixture was heated at reflux under argon for 1 h. After cooling to room temperature, the reaction was washed with water (100 mL), brine (100 mL), dried over MgSO_4_, filtered, and the solvent removed to give a dark yellow oil. The crude product was purified by column chromatography over silica (eluent: hexane) to the product as yellow oil (5.0 g, 56%). ^1^H NMR (400 MHz, CDCl_3_) *δ* 8.79 (d, *J* = 6.4 Hz, 1H), 8.36 (s, 1H), 7.77 (d, *J* = 6.4 Hz, 1H), 2.64 (t, *J* = 7.4 Hz, 2H), 1.56–1.67 (m, 2H), 1.22–1.29 (m, 18H), 0.91 (t, *J* = 7.4 Hz, 3H). ^13^C NMR (100 MHz, CDCl_3_) *δ* 153.0, 140.1, 124.1, 117.9, 34.9, 31.9, 30.4, 29.7, 29.6, 29.5, 29.4, 29.3, 22.8, 14.2. HRMS (EI) calculated for [C_16_H_28_Te] 350.1253; found 350.1254.

#### 2,5-Dibromo-3-dodecylselenophene

To a solution of 3-dodecylselenophene (2 g, 6.67 mmol) in THF (40 mL) at 0 °C was added *N*-bromosuccinimide (2.4 g, 13.47 mmol) in six portions over 40 min. The resulting solution was stirred at room temperature in the dark for 16 h. The solvent was removed under reduced pressure, and the resulting residue was dissolved in ethyl acetate (45 mL) and washed with water (2 × 25 mL). The aqueous layers were combined and further extracted with ethyl acetate. The combined organic layers were washed with brine (30 mL), dried over MgSO_4_, filtered and concentrated under reduced pressure. The crude oil was filtered through a plug of silica (eluent: petroleum ether 40–60 °C) to afford a colorless oil (2.8 g, 92%). ^1^H NMR (400 MHz, CDCl_3_) *δ* 7.0 (s, 1H), 2.51 (t, *J* = 7.8 Hz, 2H), 1.48–1.55 (m, 2H), 1.24–1.30 (m, 18H), 0.92 (t, *J* = 6.7 Hz, 3H). ^13^C NMR (100 MHz, CDCl_3_) *δ* 144.9, 134.2, 113.6, 111.1, 31.9, 30.7, 29.7, 29.6, 29.4, 29.2, 22.8, 14.2. HRMS (EI) calculated for [C_16_H_26_SeBr_2_] 455.9566; found 455.9565.

#### 2,5-Dibromo-3-dodecyl tellurophene

To a solution of 3-dodecyl tellurophene (0.5 g, 1.44 mmol) in dry ether (10 mL) at 0 °C was added *n*-BuLi (2.9 mL of a 2.5 M solution in hexanes, 7.25 mmol) dropwise over 10 min. The resulting solution was stirred at room temperature for 1 h and then refluxed for 4 h. The reaction mixture was then cooled to –50 °C and 1,2-dibromotetrachloroethane (1.17 g, 3.6 mmol) was added portionwise over 5 min. The reaction mixture was allowed to warm to room temperature overnight. The crude product was diluted with ether (100 mL) and the solid material was removed by filtration. The organic layer was washed with water (100 mL), brine (100 mL), dried over MgSO_4_, filtered and the solvent was removed under vacuum. The crude product was purified by column chromatography over silica (eluent: hexane) to afford a yellow oil (400 mg, 55%). ^1^H NMR (400 MHz, CDCl_3_) *δ* 7.43 (s, 1H), 2.56 (t, *J* = 7.8 Hz, 2H), 1.51–1.59 (m, 2H), 1.29–1.36 (m, 18H), 0.91 (t, *J* = 6.7 Hz, 3H). ^13^C NMR (100 MHz, CDCl_3_) *δ* 150.9, 141.9, 106.4, 103.7, 32.5, 31.9, 29.7, 29.6, 29.5, 29.4, 29.3, 29.2, 27.2, 22.7, 14.2. HRMS (EI) calculated for C_16_H_26_Br_2_Te 505.9464; found 505.9445.

### General polymerisation procedure

To the appropriate 2,5-dibromo-3-dodecyl monomer in a microwave vial was added Pd(PPh_3_)_4_ (1 mol%), toluene or chlorobenzene (0.6 mL) and equal molar amount of (*E*)-1,2-bis-(tributylstannyl)ethylene. The resultant mixture was degassed for 30 min with argon and securely sealed. The glass vial was either placed into an oil bath and was heated at 110 °C for 24 h when using toluene or placed into a microwave at 120 °C for 5 min, 140 °C for 5 min and 180 °C for 30 min when using chlorobenzene.

All the polymers where purified as follows: the polymerisation was cooled to room temperature, and added dropwise into a rapidly stirring mixture of methanol (200 mL)/concentrated hydrochloric acid (2 mL) and allowed to stir for 2 h. The polymeric material was then filtered under reduced pressure into a cellulose thimble and was purified by Soxhlet extraction with methanol (12 h) acetone (12 h) and hexane (12 h) in that order. The remaining, polymer was dissolved in chloroform and precipitated into methanol, filtered and dried under vacuum to achieve the desired polymer.

#### P3TV-C12

P3TV-C12 was synthesized from 2,5-dibromo-3-dodecylthiophene (0.25 g, 0.61 mmol), (*E*)-1,2-bis(tributylstannyl)ethylene (370 mg, 0.61 mmol), Pd(PPh_3_)_4_ (1 mol%, 7 mg) using the microwave conditions in chlorobenzene (0.6 mL) to afford the desired polymer after drying under vacuum (85 mg, chloroform fraction, 50%). GPC (chlorobenzene at 80 °C): *M*_w_ = 16 000, *M*_n_ = 10 100 g mol^–1^. UV-Vis *λ*_max_ (dilute chlorobenzene solution): 566 nm, *λ*_max_ (film): 572 nm. ^1^H NMR (500 MHz, C_2_D_2_Cl_4_ at 130 °C) *δ* 6.88–7.03 (br, 3H), 2.72 (br, 2H), 1.74 (br, 2H), 1.38 (br, 18H), 0.97 (br, 3H).

#### P3SV-C12

P3SV-C12 was synthesized from 2,5-dibromo-3-dodecylselenophene (0.25 g, 0.55 mmol), (*E*)-1,2-bis(tributylstannyl)ethylene (333 mg, 0.55 mmol), Pd(PPh_3_)_4_ (1 mol%, 6.3 mg) using the microwave conditions in chlorobenzene (0.6 mL) to afford the desired polymer after drying under vacuum (94 mg, chloroform fraction, 53%). GPC (chlorobenzene at 80 °C): *M*_w_ = 24 000, *M*_n_ = 12 000 g mol^–1^. UV-Vis *λ*_max_ (dilute chlorobenzene solution): 609 nm, *λ*_max_ (film): 613 nm. ^1^H NMR (500 MHz, C_2_D_2_Cl_4_ at 130 °C) *δ* 6.87–7.05 (br, 3H), 2.68 (br, 2H), 1.72 (br, 2H), 1.38 (br, 18H), 0.97 (br, 3H).

#### P3TeV-C12

P3TeV-C12 was synthesized from 2,5-dibromo-3-dodecyltellurophene (0.16 g, 0.31 mmol), (*E*)-1,2-bis(tributylstannyl)ethylene (188 mg, 0.31 mmol), Pd(PPh_3_)_4_ (1 mol%) in toluene (0.5 mL) to afford the desired polymer after drying under vacuum (66 mg, chloroform fraction, 57%). For this polymer the chloroform was heated to help dissolution before precipitation into methanol. GPC (chlorobenzene at 80 °C): *M*_w_ = 24 000, *M*_n_ = 10 000 g mol^–1^. UV-Vis *λ*_max_ (dilute chlorobenzene solution): 637 nm, *λ*_max_ (film): 652 nm. ^1^H NMR (500 MHz, C_2_D_2_Cl_4_ at 130 °C) *δ* 7.41–6.68 (br, 3H), 2.67 (br, 2H), 1.70 (br, 2H), 1.37 (br, 18H), 0.97 (br, 3H).

### Transistor fabrication and testing

#### BG/TC OFET general procedure

Bottom gate/top contact devices were fabricated on heavily doped *n*^+^-Si (100) wafers with 400 nm-thick thermally grown SiO_2_. The Si/SiO_2_ substrates were treated with OTS to form a self-assembled monolayer. The polymer was dissolved in 1,2,4-trichlorobenzene (TCB) (5 mg mL^–1^) and spin cast at 2000 rpm from a hot solution for 60 s before being annealed at 150 °C for 30 min. Au (40 nm) source and drain electrodes were deposited onto the polymer film under vacuum through shadow masks. The channel width and length of the transistors are 1000 μm and 40 μm, respectively. Mobility was extracted from the slope of *I*_D_^1/2^*vs. V*_G_.

#### BG/BC devices

BG/BC devices were fabricated on a thermally grown 300 nm layer of silicon dioxide as the dielectric and the source–drain was photolithography patterned. The substrates were thoroughly cleaned in an ultrasonic bath with acetone, de-ionised water and isopropyl alcohol and exposed to UV-light for ∼20 min. This was followed by surface treatment of the substrate using octadecyltrichlorsilane (OTS) to form a self-assembled monolayer. Solutions were deposited as above and annealed at 150 °C for 30 min.

#### TG/BC devices

TG/BC devices were fabricated on glass with Au-PFBT electrodes, CYTOP dielectric and Al gate. Polymer films were spin cast from hot solutions at 2000 rpm and annealed at 120 °C for 15 min. Polymers were dissolved in TCB (5 mg mL^–1^) and stirred at 160 °C overnight.

## Results and discussion

There are limited reports for the preparation of 3-alkyltellurophenes, and here we based our synthesis upon slight modifications of the previous reports.^22^ In order to enable ready comparison to the thiophene and selenophene–vinylene co-polymers, which were both synthesized form the respective 2,5-dibromo-3-alkylchalcogenophene monomers by polymerization with (*E*)1,2-bis(tributylstannyl)ethylene, the obvious starting material was 2,5-dibromo-3-dodecyltellurophene. However all of our attempts at electrophilic bromination of 3-dodecyltellurophene with either NBS or elemental bromine proved problematic. Complex inseparable mixtures were obtained, which we attributed to bromination of the Te atom,^29^ followed by decomposition of the resulting heterocycle. This could be avoided by di-lithiation of 3-dodecyl tellurophene with excess *n*-BuLi at room temperature followed by the low temperature addition of 1,2-dibromotetrachlorethane to afford 2,5-dibromo-3-dodecyltellurophene in 55% yield as a yellow oil. The structure of the product was confirmed by ^1^H and ^13^C NMR and high-resolution mass-spectrometry. The product was stable, with no signs of degradation seen upon storage at 5 °C over several months.

**Scheme 1:**
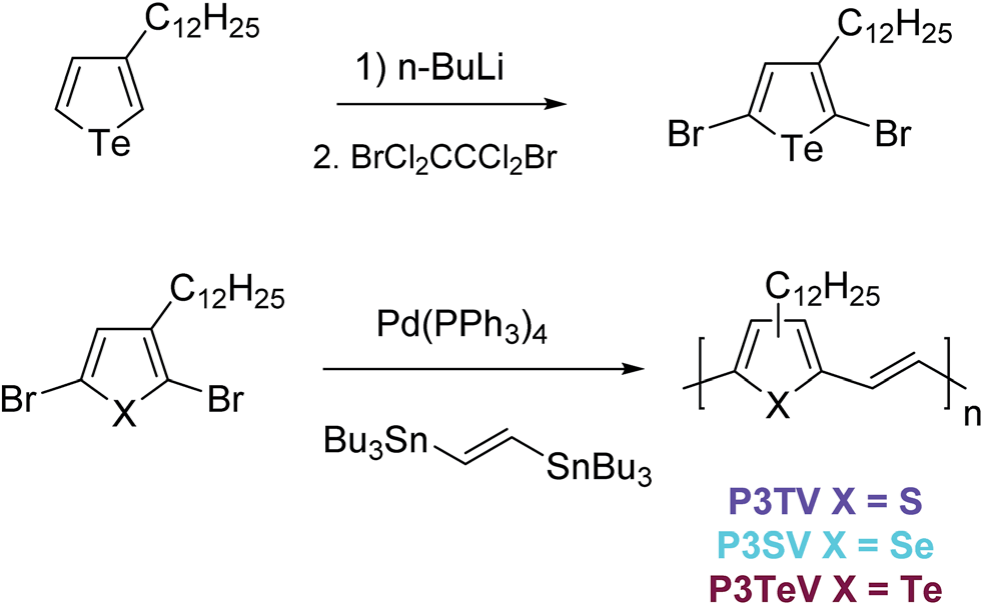
Synthesis of 2,5-dibromo-3-dodecyltellurophene and polymerization to afford **P3TV**, **P3SV** and **P3TeV**.

With all three dibrominated monomers in hand, the three polymers **P3TV**, **P3SV** and **P3TeV** were synthesized by Stille coupling between the appropriate 2,5-dibromo-3-dodecyl monomers and (*E*)1,2-bis(tributylstannyl)ethylene (Scheme 1). Polymers **P3TV** and **P3SV** where synthesized using microwave assisted heating in chlorobenzene, whereas polymer **P3TeV** was synthesized using conventional heating in toluene. Attempted microwave polymerization of **P3TeV** resulted in insoluble polymers, which we attributed to the formation of insoluble high molecular weight material. The crude polymers were all purified by precipitation and Soxhlet extraction with methanol and acetone to remove catalyst residues and low molecular weight oligomers, followed by precipitation of a chloroform solution of the polymer into methanol to afford the polymers in good yield. Clear differences were observed in the qualitative solubility of the polymers, which decreased in the trend **P3TV** > **P3SV** > **P3TeV**. The **P3TeV** polymer was only slightly soluble at room temperature in chlorinated solvents like chlorobenzene or chloroform, but could be dissolved upon prolonged heating. Nevertheless it had a high tendency to precipitate upon cooling, resulting in difficulties in the formation of smooth films.

The molecular weight (*M*_n_ and *M*_w_) and dispersity index (*Ð*) of the copolymers were determined by gel permeation chromatography (GPC) relative to polystyrene standards in hot chlorobenzene at 80 °C. The three polymers exhibit similar average molecular weights (*M*_w_) in the range of 16–24 kDa, although there is some variation in the dispersity between the polymers (Table 1). It is worth highlighting that GPC calibrated against flexible polystyrene standards often significantly overestimates the molecular weight of semi-flexible conjugated polymers, suggesting these are likely upper estimates of the actual values.^30^,^31^

**Table 1:** Molecular weights, optical properties and experimental energy levels of **P3TV**, **P3SV** and **P3TeV**

Polymers	*M*_n_ ^*a*^ (kDa)	*M*_w_ ^*a*^ (kDa)	*Ð* ^*a*^	*λ*_max_ soln [nm]	*λ*_max_ film [nm]	*λ*_onset_ film [nm]	HOMO (PESA)^*b*^ [eV]	LUMO^*c*^ [eV]	HOMO (CV)^*d*^ (*E*_ox_, onset V)	*E* opt g ^*e*^ [eV]
**P3TV**	10.1	16	1.58	566	572	725	–4.93	–3.22	–5.21 (0.70)	–1.71
**P3SV**	12	24	2	609	613	800	–4.95	–3.40	–5.17 (0.66)	–1.55
**P3TeV**	10	24	2.4	637	652	875	–4.89	–3.50	–5.08 (0.57)	–1.42

^*a*^Determined by GPC and reported as their polystyrene equivalents.

^*b*^Determined as a thin film by UV-PESA error (±0.05 eV).

^*c*^Estimated using HOMO^*b*^ – *E*optg.

^*d*^CV measurements performed on as cast polymer films on a Pt working electrode in CH_3_CN with 0.1 M TBAPF_6_. The scan rate was 50 mV s^–1^. *E*_HOMO_ = –(*φ*^onset^ ox – *φ*_Fc/Fc_^+^ + 4.8).

^*e*^Determined by onset of optical absorption of thin film deduced from the equation, *E*optg = 1240/*λ*_onset_.

**Fig. 1:**
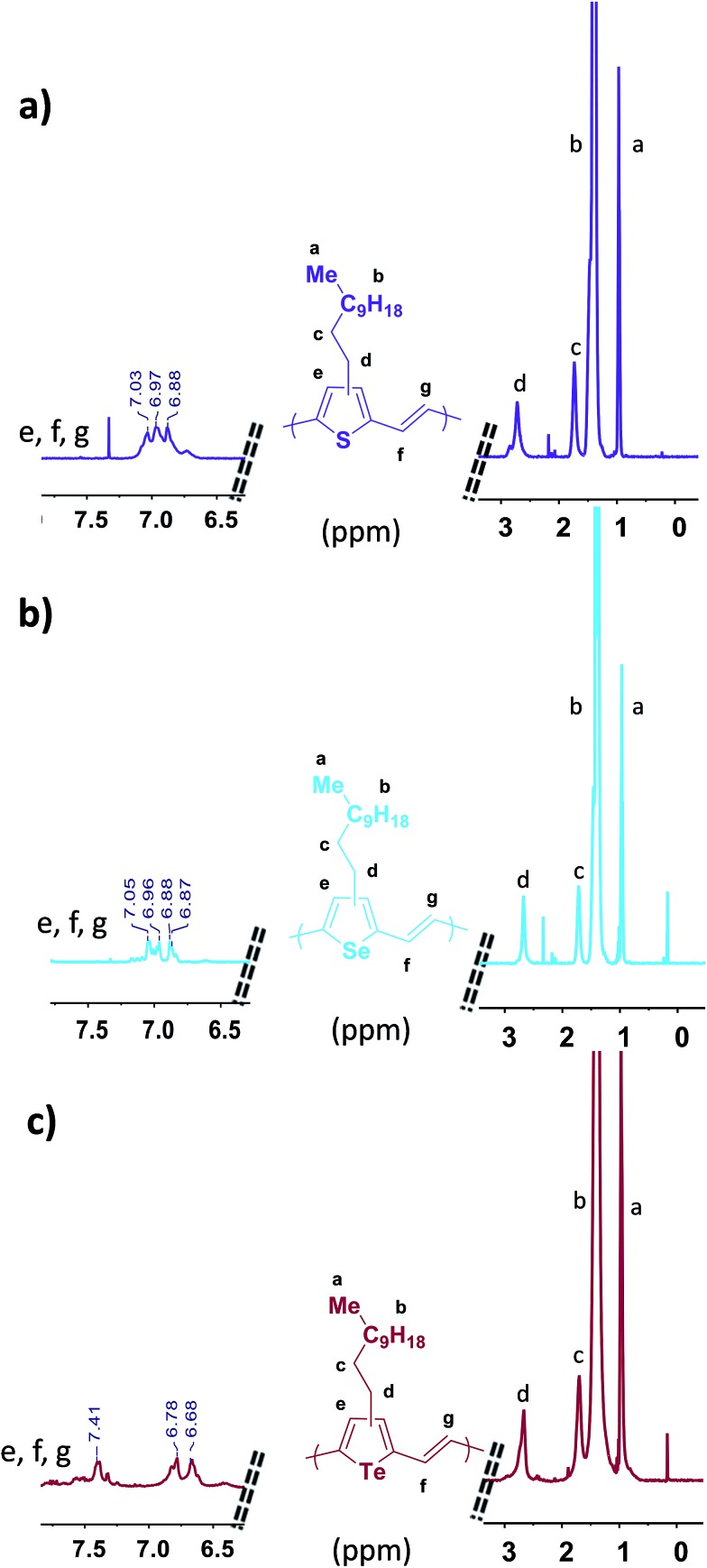
^1^H NMR spectra of the three polymers; (a) **P3TV**, (b) **P3SV** and (c) **P3TeV** (500 MHz, C_2_D_2_Cl_4_, 130 °C).

The chemical structure of the three polymers were investigated using ^1^H NMR spectroscopy in 1,1,2,2-tetrachloroethane-d_2_ at 130 °C (Fig. 1). Loss of resolution and broadening of the spectra can be seen due to the high degree of polymer aggregation in solution even at 130 °C. Nevertheless, the signals corresponding to the aromatic and vinyl protons of **P3TV**, **P3SV** and **P3TeV** can be resolved in the range of *δ* 6.6 to 7.5 ppm. Interestingly, **P3TV** and **P3SV** polymers show similar and overlapping chemical shifts at *δ* 6.87 to 7.05 ppm for the aromatic and vinylene protons as previously observed,^25^,^32^ whereas **P3TeV** shows significant differences, with the aromatic proton shifted downfield to a broad multiplet around 7.41 ppm, and the vinylene protons are shifted upfield to a doublet 6.78–6.68 ppm. The broadness of the tellurophene signal is probably related to end group effects due to the low molecular weight. The methylene protons for all polymers all fall as a broad peak in a similar region around *δ* 2.72–2.65 ppm. Studies on **P3TV**'s of well-defined regularity show that the methylene region is not very sensitive to the regiochemistry of the coupling, and that the aromatic region is more informative.^33^ However it is difficult to assess the regioregularity of the polymers due to the broadness of the aromatic peaks in the current case, but we can say all polymers are similar. Although early reports of PTV's by Stille polymerization suggested a regioregularity around 85–90%,^32^ later reports^33^ suggest the polymers are regiorandom. As noted earlier, low regioregularity is not expected to prevent backbone planarization in this case.

**Fig. 2:**
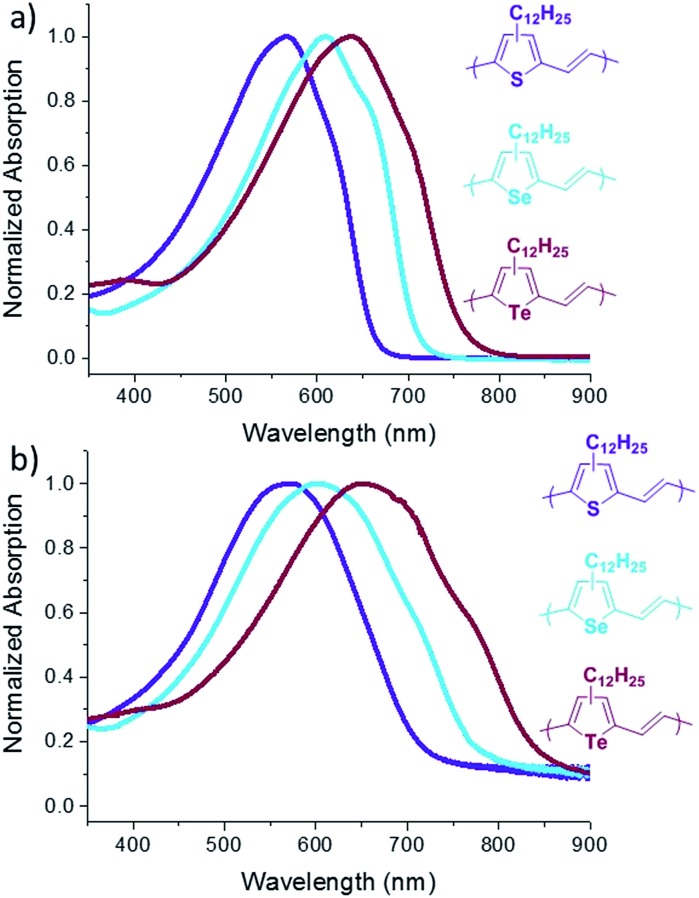
UV-Vis spectra of **P3TV**, **P3SV** and **P3TeV** in dilute chlorobenzene (a) and in thin films (b).

The absorption spectra of **P3TV**, **P3SV** and **P3TeV** in solution and as drop cast films are shown in Fig. 2 and the data is summarized in Table 1. There is a clear trend across the series, with the spectra in both solution and thin film red-shifting as the heteroatom is changed, hence moving from sulfur (**P3TV**) to tellurium (**P3TeV**) results in a red shift of 71 nm in solution and 80 nm in film, whereby the polymer containing the tellurium atom shows the largest red-shift absorption in both solution and film. In solution the spectra all display similar shape, with an absorption maxima (*λ*_max_) at 566, 609 and 637 nm for **P3TV**, **P3SV** and **P3TeV** respectively. There is also a pronounced shoulder at longer wavelengths for all polymers, this is typically associated with vibronic coupling and suggests some degree of order for the polymers, even in solution. Upon film formation the *λ*_max_ of all polymers red-shifts slightly (6–15 nm) and broaden significantly. The vibronic shoulder appears to become more pronounced on moving down the periodic table, becoming especially prominent for **P3TeV**. The optical band gaps of the three polymers as estimated from the onset absorption of the thin film are **P3TV** = 1.71 eV, **P3SV** = 1.55 eV and **P3TeV** = 1.42 eV, and follow a similar trend to the shift in *λ*_max_. Although the vibronic shoulders are indicative of some short range aggregation in the solid state, investigation of the polymers by differential scanning calorimetry (DSC) was uninformative (ESI†), with either no peaks observed (**P3SV**, **P3TeV**) or only very broad transitions (**P3TV**) upon cycling to 200 °C. Heating above 200 °C was previously noted to result in irreversible changes in phase behavior.^25^ The lack of peaks in the current examples *versus* those previously observed for **P3TV** and **P3SV**^25^ is likely due to the lower molecular weight and regioregularity of the current materials *versus* previous examples.

**Fig. 3:**
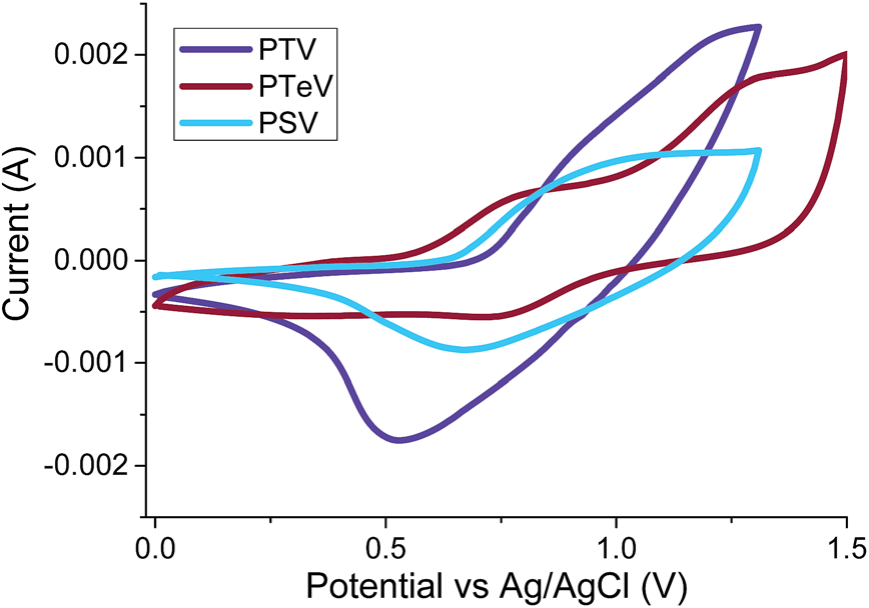
Thin-film cyclic voltammograms of **P3TV**, **P3SV** and **P3TeV** on Pt electrodes in CH_3_CN/0.1 M [*n*Bu_4_N]^+^[PF_6_]^–^ at 50 mV s^–1^.

The oxidation potentials of the polymers were obtained using cyclic voltammetry (CV) measurements of drop-cast polymers films on a platinum working electrodes. The cyclic voltagrams are shown in Fig. 3 and show quasi-reversible oxidation waves in the case of **P3TV** and **P3SV**. In the case of **P3TeV** a fully reversible oxidation is observed, with a second reversible oxidation observed at higher voltages (*E*_ox2_ onset 1.04 V). We were not able to observe reproducible reduction for any polymer under our test conditions. The HOMO energies for **P3TV**, **P3SV** and **P3TeV** were estimated by the onset of oxidation (*E*_ox_ onset, see Table 1) against Ag/AgCl reference, assuming the ferrocene/ferrocenium reference redox system is 4.8 eV below the vacuum level. The ionization potentials were also measured as thin films using photoelectron spectroscopy (PESA, Fig. S1–S3†). The absolute values measured by either technique are different, but there is reasonable agreement in the trends observed. Thus both CV and PESA find similar HOMO values for the **P3TV** and **P3SV**, with the differences within the error of either technique, suggesting that the substitution of sulfur for selenium has a relatively minor influence of the oxidation potential. The clear difference in optical band gap therefore suggests a greater perturbation to the LUMO, with the decrease in band gap suggesting a stabilizing influence on the LUMO on moving to the larger heteroatom.^34^,^35^ On moving down the periodic table, both PESA and CV suggest the HOMO of **P3TeV** moves closer to the vacuum level compared to the sulfur or selenium analogues. The increase in the HOMO level (*ca.* 0.05–0.1 eV depending on technique) is substantially less than the reduction in optical band gap, suggesting that the perturbation to the LUMO is greater than the HOMO. The increase in the HOMO level observed is in agreement with other reports on tellurophene containing polymers,^10^ and with the observation that the aromaticity of tellurophene is low in comparison to thiophene and selenophene as a result of the reduced overlap between the larger heteroatom and the adjacent carbon based π orbitals.^36^

Finally the charge carrier mobilities of the polymers were investigated in field effect transistors, using a variety of device architectures including bottom gate, bottom contact (BG/BC), bottom gate, top contact (BG/TC) and top gate, bottom contact (TG/BC). In all cases the processing of the films was problematic due to the moderate solubility and this was a particular problem for **P3TeV** which rapidly precipitated from solution on cooling. Although heating of the substrate prior to spin-casting helped, the device yield remaining low. **P3TV** was also problematic due to wetting issues, particularly for BG/BC devices. Nevertheless working devices could be obtained for all polymers in at least two of the device architectures, and the data is summarized in Table 2, with individual transfer characteristics in the ESI.† All polymers were p-type semiconductors with no evidence of electron transport observed in these configurations. The devices showed some hysteresis between the forward and reverse scans in most cases, which may relate to the difficulty in film formation. In terms of mobility, relatively modest saturated mobilities were observed for all polymers, in agreement with the previous reports of **P3TV** and **P3SV** prepared by Stille polymerization.^25^,^27^ Direct comparisons between the polymers are complicated by the film forming issues, but it is clear that the selenophene polymer consistently displays the highest transistor mobility across all devices. The incorporation of tellurium does not seem to confer any beneficial increase in mobility, despite the large size of Te and the possibility of interchain Te···Te interactions.^37^

**Table 2:** Average saturated field effect mobilities in various transistor architectures (TG/BG)

Polymer	Field effect mobility (cm^2^ V^–1^ s^–1^)		
	TG/BC	BG/BC	BG/TC
**P3TV**	5 × 10^–4^	—	1.8 × 10^–3^
**P3SV**	0.05	0.02	3.6 × 10^–3^
**P3TeV**	1 × 10^–4^	0.001	—

To the best of our knowledge there is only one report of the transistor properties of a tellurophene homo polymer, in which the performance of regioregular P3ATe with varying sidechain lengths is reported with moderate mobility.^38^ There are even fewer examples comparing the performance of polymers with different chalcogenophenes and all of these examples have been co-polymers in which the tellurophene comprises a relatively minor fraction of the backbone. In these limited reports, the inclusion of Te has been shown to improve mobility over S and Se.^13^,^15^,^17^ In the case of vacuum deposited small molecules, Te inclusion has also resulted in a reduction in charge carrier mobility compared to S or Se.^39^ In our case any beneficial influence of the Te on mobility in terms of enhanced molecular contacts seems countered by the reduced solubility resulting in poor film forming ability. These results suggest attempts to fully harness the potential of all tellurophene containing polymers need to pay close attention to sidechain engineering to enhance solubility.

## Conclusions

In conclusion we have reported the synthesis of 2,5-dibromo-3-dodecyltellurophene and co-polymerized it with (*E*)1,2-bis(tributylstannyl)ethylene to afford the first tellurophene vinylene co-polymer. Analogous thiophene and selenophene containing polymers were prepared by the same methodology to produce polymers of similar molecular weight and regioregularity. Increasing the size of the heteroatom results in a reduction in solubility as well as a reduction in the optical band gap, mainly as a result of the stabilization of the polymer LUMO level. Field effect transistors in a variety of device architectures show that the selenophene containing polymers consistently exhibit the highest mobility, whereas the low solubility of the tellurophene containing polymer inhibited device performance, highlighting the requirement to carefully tune sidechains for solubility in the case of heavy atom containing polymers.

## Supplementary Material

Supplementary information
