# Supplementary material for: Influence of the heteroatom on the optoelectronic properties and transistor performance of soluble thiophene-, selenophene- and tellurophene–vinylene copolymers
Source: Chem Sci. 2015 Nov 2;7(2):1093–9. doi: 10.1039/c5sc03501e (PMC5954972; doi:10.1039/c5sc03501e)
Supplement: Supplementary file 1 [file SC-007-C5SC03501E-s001.pdf]

Electronic Supplementary Material (ESI) for Chemical Science.
This journal is © The Royal Society of Chemistry 2015

Influence of the heteroatom on the properties and transistor performance of soluble thiophene-, selenophene- and tellurophene-vinylene copolymers

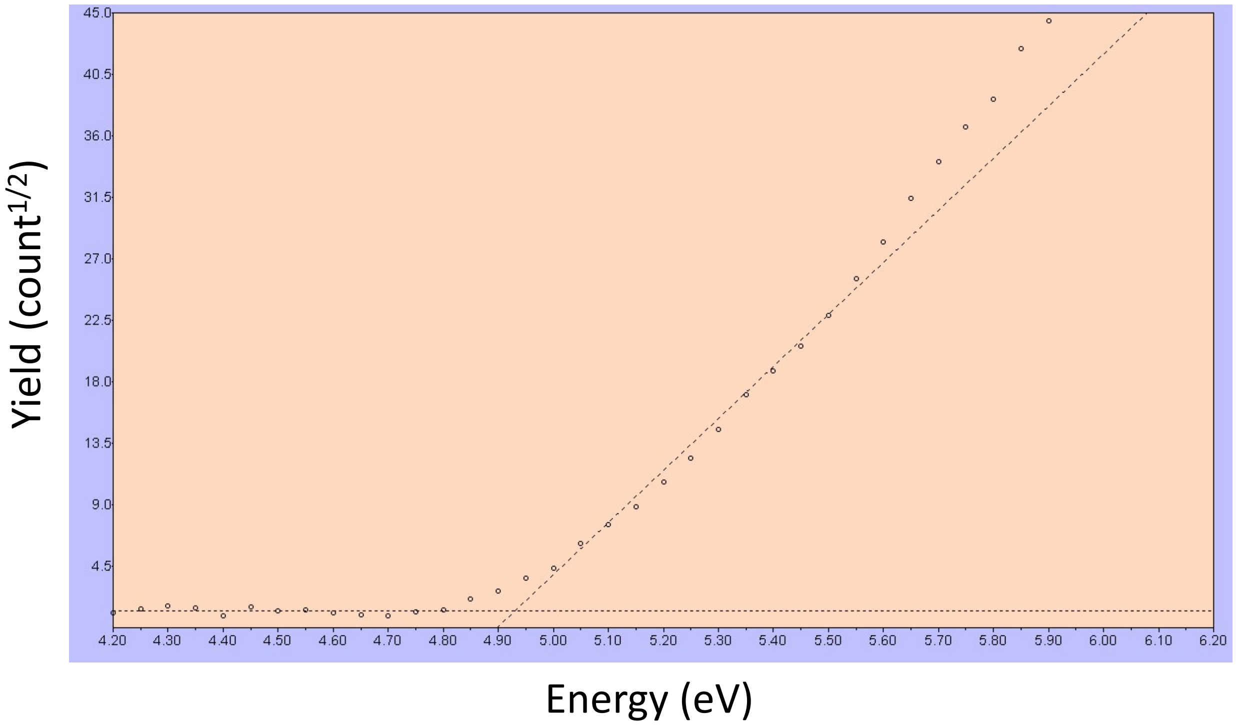

Figure S1. Photo Electron Spectroscopy in Air (PESA) spectra of a thin film of **P3TV.** The ionization potential was calculated by extrapolating a fit of the square root of the photoelectron emission yield as a function of excitation photon energy.

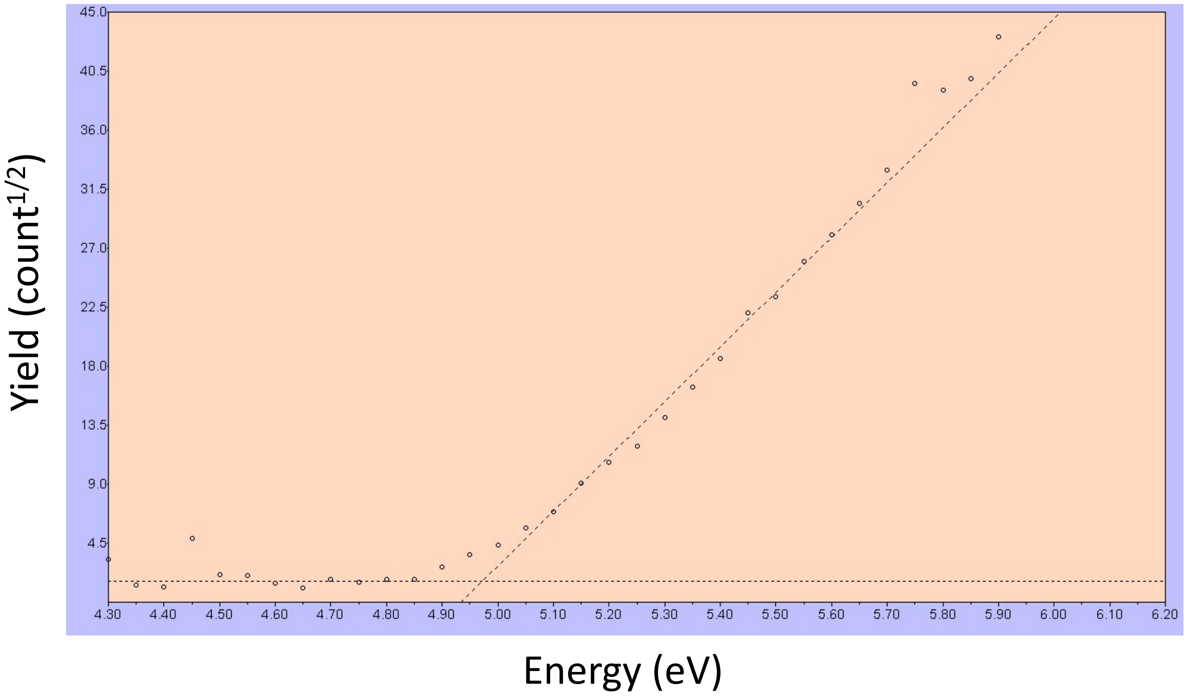

Figure S2. Photo Electron Spectroscopy in Air (PESA) spectra of a thin film of **P3SV**. The ionization potential was calculated by extrapolating a fit of the square root of the photoelectron emission yield as a function of excitation photon energy.

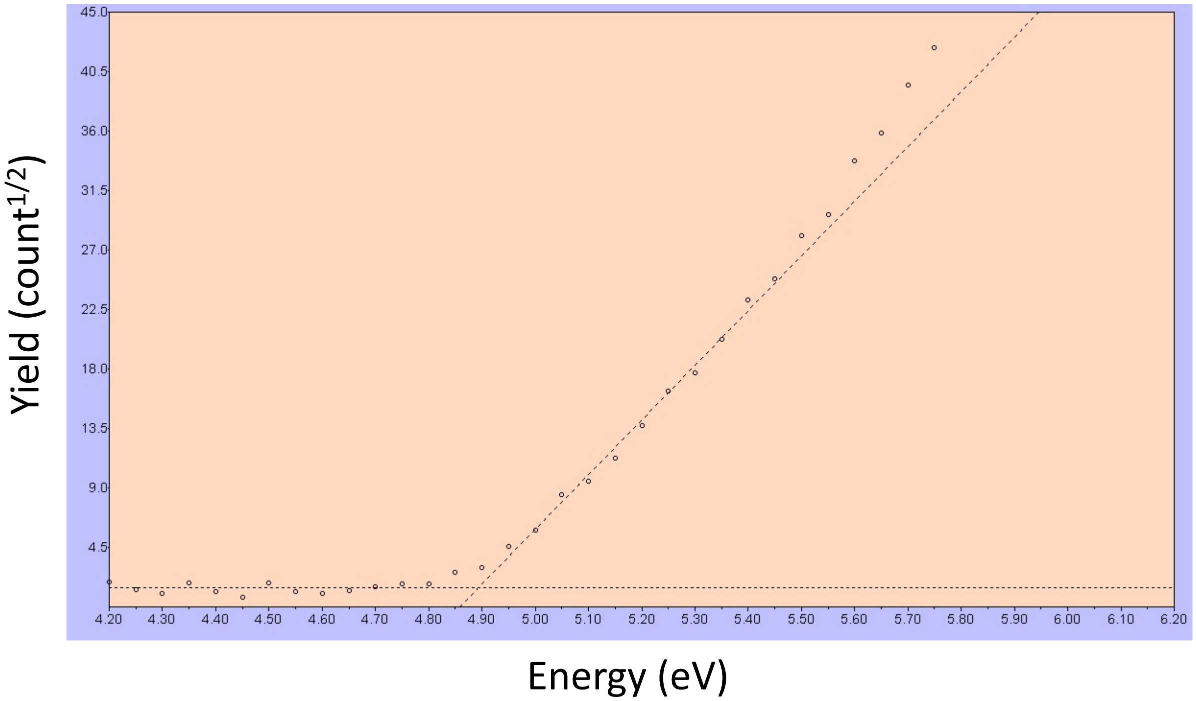

Figure S3. Photo Electron Spectroscopy in Air (PESA) spectra of a thin film of **P3TeV.** The ionization potential was calculated by extrapolating a fit of the square root of the photoelectron emission yield as a function of excitation photon energy.

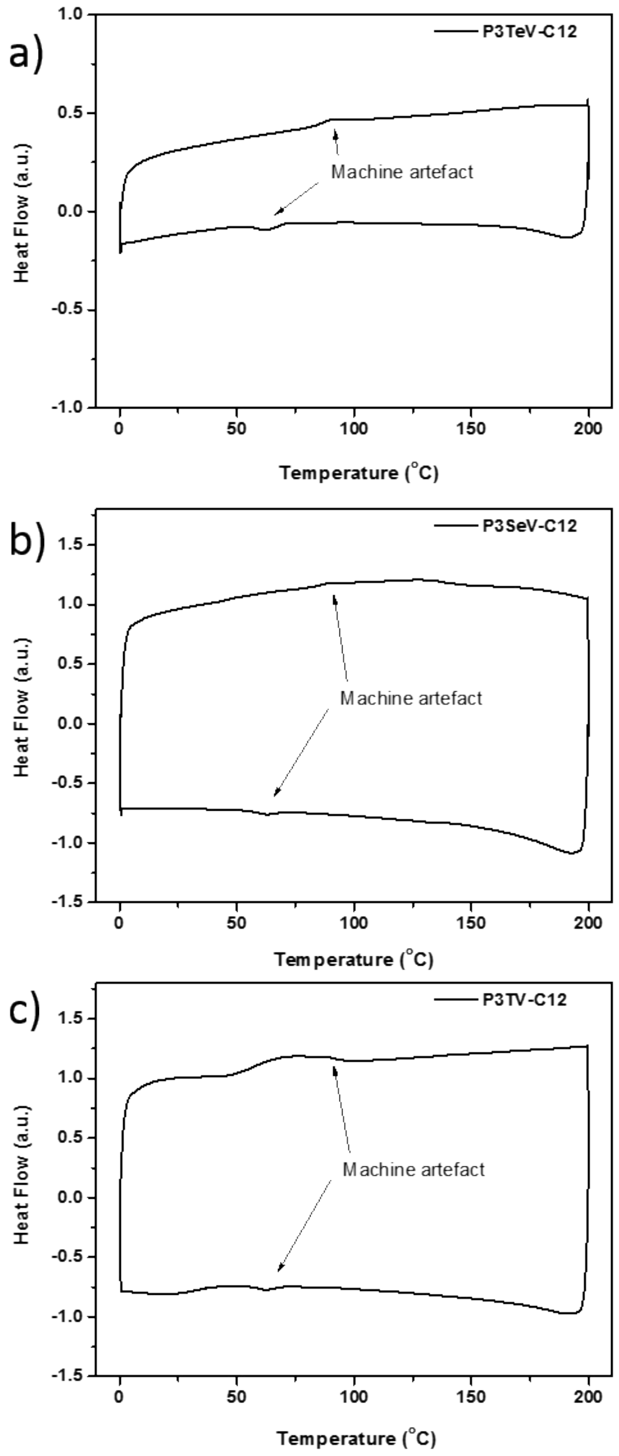

Figure S4. DSC plots (endo up) of a) P3TeV b) PSV and c) P3TV recorded at a heating and cooling rate of 10 ºC min^{-1} under nitrogen (second cycle).

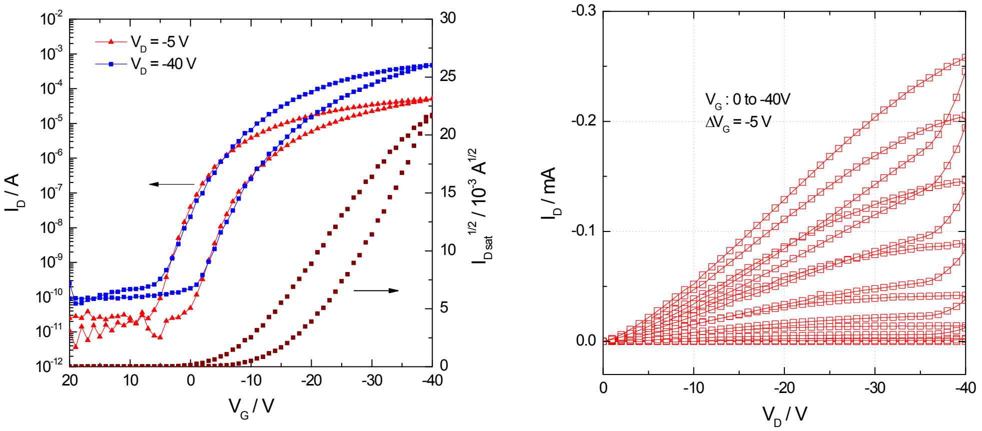

Figure S5. BG/BC transfer and output plots of **P3SV**.

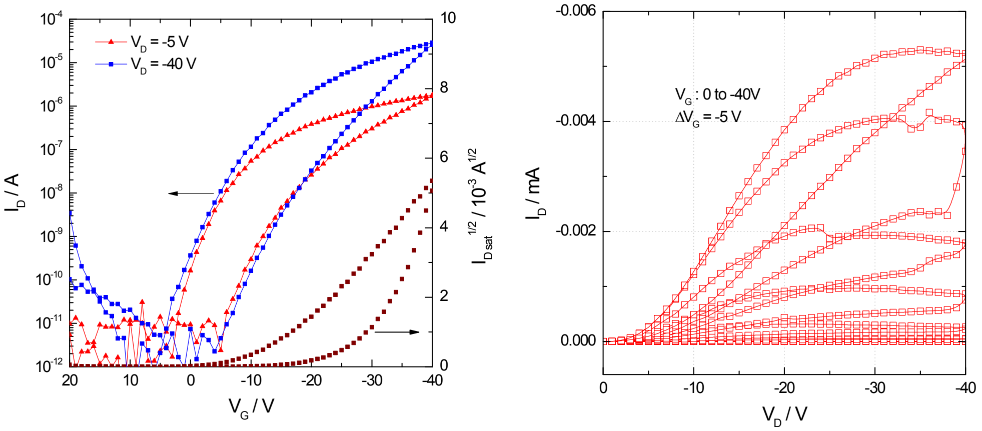

Figure S6. BG/BC transfer and output plots of **P3TeV**.

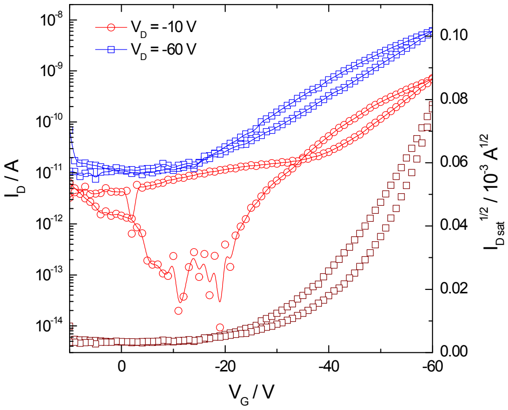

Figure S7. TG/BC transfer plots **P3TV** (channel length = 30 μm, width = 1000 μm)

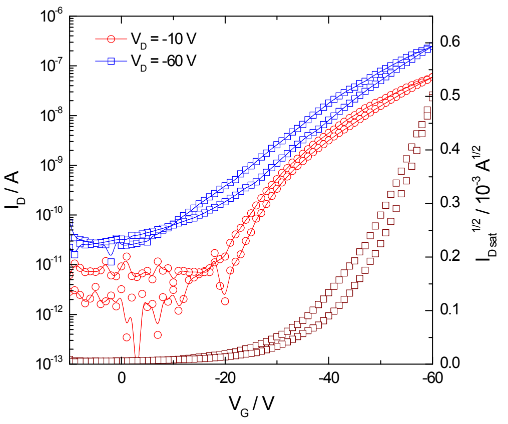

Figure S8. TG/BC transfer plots **P3SV** (channel length = 50 μm, width = 1000 μm)

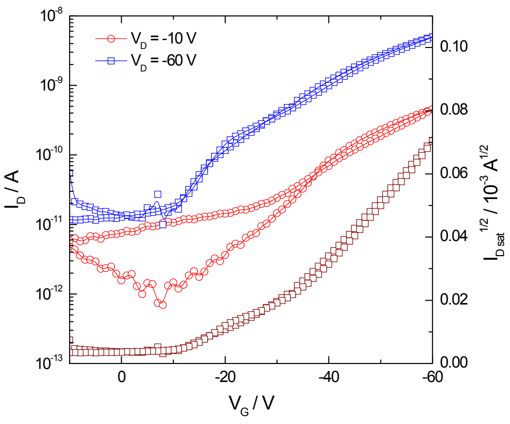

Figure S9. TG/BC transfer plots **P3TeV** (channel length = 50 μm, width = 1000 μm)

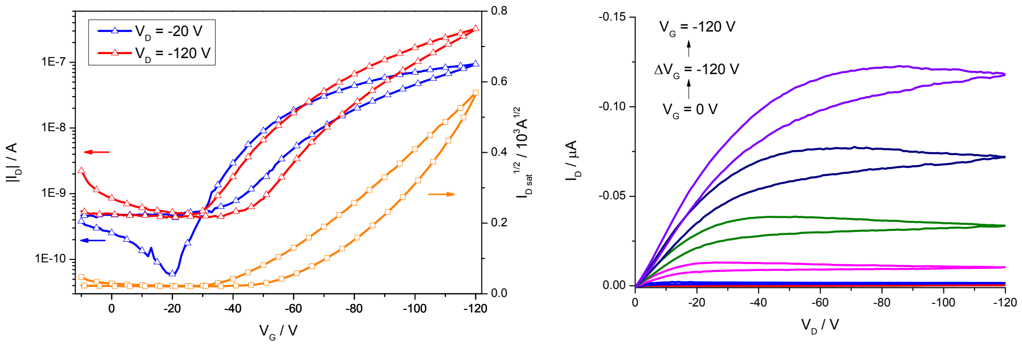

Figure S10. BG/TC transfer and output plots **P3TV**

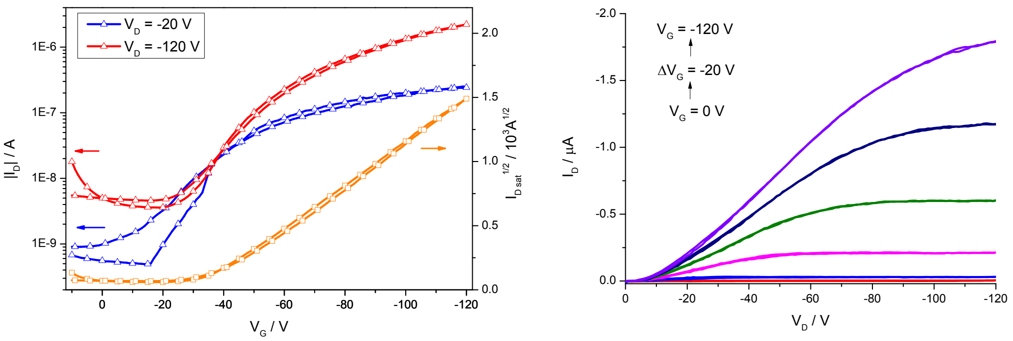

Figure S11. BG/TC transfer and output plots **P3SV**
